# Highly Dispersed RuOOH Nanoparticles on Silica Spheres: An Efficient Photothermal Catalyst for Selective Aerobic Oxidation of Benzyl Alcohol

**DOI:** 10.1007/s40820-020-0375-9

**Published:** 2020-01-27

**Authors:** Qilin Wei, Kiersten G. Guzman, Xinyan Dai, Nuwan H. Attanayake, Daniel R. Strongin, Yugang Sun

**Affiliations:** Department of Chemistry, Temple University, 1901 North 13th Street, Philadelphia, PA 19122 USA

**Keywords:** Ultrasmall RuOOH nanoparticles, Photothermal catalyst, Selective aerobic oxidation, Light antenna effect, Light scattering resonance

## Abstract

Photothermal catalysis represents a promising strategy to utilize the renewable energy source (e.g., solar energy) to drive chemical reactions more efficiently. Successful and efficient photothermal catalysis relies on the availability of ideal photothermal catalysts, which can provide both large areas of catalytically active surface and strong light absorption power simultaneously. Such duplex requirements of a photothermal catalyst exhibit opposing dependence on the size of the catalyst nanoparticles, i.e., smaller size is beneficial for achieving higher surface area and more active surface, whereas larger size favors the light absorption in the nanoparticles. In this article, we report the synthesis of ultrafine RuOOH nanoparticles with a size of 2–3 nm uniformly dispersed on the surfaces of silica (SiO_*x*_) nanospheres of hundreds of nanometers in size to tackle this challenge of forming an ideal photothermal catalyst. The ultrasmall RuOOH nanoparticles exhibit a large surface area as well as the ability to activate adsorbed molecular oxygen. The SiO_*x*_ nanospheres exhibit strong surface light scattering resonances to enhance the light absorption power of the small RuOOH nanoparticles anchored on the SiO_*x*_ surface. Therefore, the RuOOH/SiO_*x*_ composite particles represent a new class of efficient photothermal catalysts with a photothermal energy conversion efficiency of 92.5% for selective aerobic oxidation of benzyl alcohol to benzylaldehyde under ambient conditions.

**Electronic supplementary material:**

The online version of this article (10.1007/s40820-020-0375-9) contains supplementary material, which is available to authorized users.

## Highlights

- Ultrasmall RuOOH nanoparticles of 2–3 nm are loaded on submicron silica spheres and capable of activating molecular oxygen.
- Photothermal conversion efficiency of the supported RuOOH nanoparticles is nearly unity.
- Photothermal effect promotes selective oxidation of benzyl alcohol under the illumination of visible light.

**Electronic supplementary material:**

The online version of this article (10.1007/s40820-020-0375-9) contains supplementary material, which is available to authorized users.

## Graphical abstract

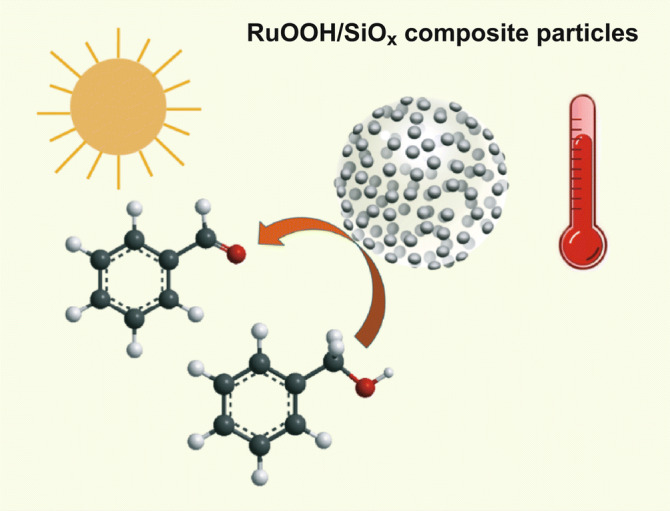

## Introduction

Selective aerobic oxidation of primary alcohols to corresponding aldehydes is of great interest in the chemical synthesis industry because of the environment-friendly and cost-effective features [1–4]. The high bond dissociation energy of α-C–H and weak oxidizing power of molecular oxygen (O_2_) lead to challenges for successful reactions under mild ambient conditions [5–8]. The promising solution relies on the use of appropriate catalysts and elevated temperatures (80–120 °C, if necessary) [1, 9]. The general principle of designing a catalyst is to reduce the size of catalyst nanoparticles to maximize their surface area, simultaneously increasing their catalytic activity and lowering the usage of catalyst materials (in particular precious metals) [10, 11]. In this work, we introduce highly dispersed RuOOH nanoparticles (NPs) with sizes of 2–3 nm that are synthesized through the simple hydrolysis of Ru(III) salt as a unique catalyst for selective aerobic oxidation of benzyl alcohol (BzOH) to benzylaldehyde (BzAD). The corresponding oxidation reaction highly depends on the partial pressure of O_2_, significantly different from the hydrated RuO_2_ and Ru(OH)_3_ nanoparticle (NP) catalysts reported in the literature, on which the oxidation reactions are independent of the partial pressure of O_2_ [12–15]. The change in reaction mechanism enables the aerobic oxidation of BzOH at mild temperatures and an additional strategy for tuning reaction rate by varying the partial pressure of O_2_.

The lowered reaction temperature makes it appropriate to use the more energy-efficient way, such as photothermal effect [16], to heat the reaction solution since the RuOOH NPs can absorb light of a broad spectral region, including visible light [17]. The extremely large surface-to-volume ratio of the ultrafine RuOOH catalyst NPs can significantly lower the usage of total RuOOH material compared to the reactions catalyzed with large-sized NPs. However, the small size of the RuOOH NPs with 2–3 nm in diameters limits their light absorption power. This challenge is tackled by loading the RuOOH catalyst NPs onto silica nanospheres (SiO_*x*_ NSs) with an average diameter of 443 nm, resulting in a significant increase in colloidal stability of the RuOOH NPs. The SiO_*x*_ NSs with the maximum geometric symmetry also represent a novel class of dielectric antennae that can produce enhanced electric fields near the particle surface upon light illumination due to the surface light scattering resonances [18]. Such an antenna effect benefits the light absorption efficiency of small nanoparticles loaded on the surfaces of the SiO_*x*_ NSs to promote light harvesting and the following energy conversion processes, such as hot-electron-driven chemical transformation [19–21] and photothermal-induced phenomena [22]. The RuOOH/SiO_*x*_ composite particles behave as a new class of photothermal catalysts for selective aerobic oxidation of primary alcohols with an energy conversion efficiency of as high as 92.5%.

## Materials and Methods

### Materials Synthesis

*Synthesis of SiO*_*x*_ *NSs* The synthesis relied on the controlled hydrolysis of tetraethyl orthosilicate (TEOS, 98%, Sigma-Aldrich) and condensation of the hydrolyzates [18]. In a typical synthesis, 19.6 mL of ammonia hydroxide solution (28–30 wt% in water, Fisher Scientific) was added to a mixture of 32 mL of deionized (DI) H_2_O and 260 mL of 190-proof ethanol. To this solution was then injected 17 mL of TEOS. The magnetic stirring at a rate of 600 rpm (revolutions per min) and the room temperature were maintained throughout the entire synthesis for 2 h. The resulting SiO_*x*_ NSs were collected by centrifugation and washed with ethanol to remove the unreacted species. The collected SiO_*x*_ NSs were dried overnight in an oven set at 60 °C.

*Surface modification of the SiO*_*x*_ *NSs* Dispersing 400 mg of the dried SiO_*x*_ NSs in 200 mL of ethanol was carried out with the assistance of ultrasonication. The dispersion was heated up to 60 °C followed by a dropwise addition of 2 mL of (3-aminopropyl)triethoxysilane (APTES, 98%, Acros Organics) at a rate of 1 mL min^−1^. The dispersion was continuously incubated at 60 °C for 8 h while a magnetic stirring was maintained. This process linked APTES to the surfaces of the SiO_*x*_ NSs. The APTES-modified SiO_*x*_ NSs were then collected by centrifugation and washed with ethanol. The powder was dried in the air at ambient conditions.

*Deposition of RuOOH NPs on the SiO*_*x*_ *NSs* 100 mg of APTES-modified SiO_*x*_ NSs was first dispersed in 50 mL of DI H_2_O. To this dispersion was injected 1 mL of an aqueous solution containing 3.09 mg (11.88 μmol) of ruthenium trichloride hydrate (RuCl_3_·*x*H_2_O, Alfa Aesar). Hydrolysis of RuCl_3_ in water deposited Ru(III) hydrolyzate on the surfaces of the SiO_*x*_ NSs that provided sufficient nucleation sites for forming nanoparticles. After 1 h, the resulting composite particles were collected by centrifugation and washed with DI H_2_O. Inductively coupled plasma atomic emission spectroscopy (ICP-OES) was used to analyze the loading of Ru(III) species on the SiO_*x*_ NSs, showing 1.1 wt% loading of Ru (III) that corresponded to 92% of the feeding precursor. The composite particles were then dried in an oven set at 60 °C for 1 h, followed by mild thermal annealing in a tube furnace set at 150 °C for 1 h. The thermal annealing was performed in the ambient air atmosphere. The resulting supported RuOOH NPs exhibit very small sizes in the range of 2–3 nm.

*Direct synthesis of RuOOH powders* An aqueous solution of APTES was prepared by adding 200 μL of APTES to 10 mL of DI H_2_O under vigorous magnetic stirring. To this solution was injected 20 mL of an aqueous solution containing 100 mg of RuCl_3_·*x*H_2_O. After the reaction lasted 1 h, the powder was collected by centrifugation and washed by DI H_2_O twice. The as-prepared freestanding RuOOH powder was dried in an oven and annealed in a furnace, as same as the RuOOH/SiO_*x*_ composite powders. When the concentration of APTES was significantly increased to 3 mL and the mass of RuCl_3_·*x*H_2_O was decreased to 30 mg, the basicity of the reaction solution enhanced to promote the rapid hydrolysis of Ru(III) and the high-concentration APTES also promoted its self-hydrolysis to form SiO_*x*_ NSs. As a result, the simultaneous hydrolysis of Ru(III) and APTES formed RuOOH/SiO_*x*_ composite particles with agglomerated RuOOH NPs of large sizes.

### Characterizations

Scanning electron microscopy (SEM) images were obtained using a field-emission microscope (FEI Quanta FEG 450) operated at an acceleration voltage of 10 kV in high vacuum mode. Transmission electron microscopy (TEM) images were taken using the JEOL JEM-1400 microscope operated at 120 kV. X-ray photoelectron spectra (XPS) were recorded using a VG Scientific 100 mm hemispherical analyzer equipped with a Physical Electronics Mg Kα X-ray source operating at 300 W. Raman spectroscopy was analyzed with a Horiba Jobin–Yvon Labram HR800 Evolution confocal Raman spectrometer using a 532-nm laser. The Olympus MPlan N 100× microscope objective was used to focus the excitation laser down to a spot size of ~ 1 μm. A 1800 g mm^−1^ grating was used to provide a spectral resolution of 0.5 cm^−1^. Diffuse reflectance spectroscopy (DRS) was analyzed with an ultraviolet–visible (UV–Vis) spectrophotometer (Thermo Scientific, Evolution 220) equipped with an integrating sphere detector.

### Selective Oxidation of BzOH Driven by Photothermal Effect

The photothermal conversion was evaluated by continuously recording the temperature variation of the dispersion of colloidal nanoparticles under photo-illumination. In a typical measurement, 3 mL of nanoparticle dispersion was filled in a 4-mL glass vial. The vial was then wrapped with a layer of glass wool to minimize heat dissipation. Illuminating the nanoparticle dispersion with an LED light (Fiber-Lite Mi-LED series Illuminator, Dolan-Jenner) of 965 mW in power increased the temperature of the dispersion. Once the LED light was turned off, the temperature of the dispersion started to drop. The variation of temperature was continuously recorded with a ThermoWorks USB Reference Thermometer connected to a computer.

The selective oxidation of BzOH (Acros Organics) was performed in the presence of the as-synthesized RuOOH/SiO_*x*_ composite particles. Typically, 20 mg of the RuOOH/SiO_*x*_ particles was dispersed in 3 mL of benzotrifluoride (BTF, Alfa Aesar) filled in a 4-mL glass vial that was sealed with a rubber septum. BTF was chosen as the solvent because of the good dispersity of the RuOOH/SiO_*x*_ particles and the reasonable solubility of BzOH and O_2_. In a typical reaction, the dispersion was first purged with an N_2_/O_2_ gas containing varying concentrations of O_2_ in N_2_ at 1 atm for 15 min. The atmosphere was then maintained above the dispersion, and the dispersion was illuminated with the LED light until the temperature became stable. To the photo-illuminated dispersion was injected 46 μL (0.444 mmol) of BzOH, triggering the selective oxidation of BzOH. The reactions in the dark were conducted by immersing the dispersion in a water bath that was used to tune the reaction temperature. The concentrations of BzOH and BzAD in reaction dispersion were analyzed by sampling aliquots of 0.2 mL dispersion. The aliquots were filtered with a 0.2-μm-pore-size filter (SEOH) to remove the RuOOH/SiO_*x*_ composite particles, and the filtrates were analyzed with a gas chromatography (GC) instrument (Agilent 7820A) equipped with an HP-5 column and a flame ionization detector (FID).

## Results and Discussion

The SiO_*x*_ NSs synthesized through the controlled sol-gel process exhibit a uniform spherical geometry and uniform size with an average diameter of 443 nm (Fig. S1). Surface modification of the SiOx NSs with APTES does not change their geometrical and dimensional uniformity. The APTES-modified SiO_*x*_ NSs expose amino groups (i.e., a type of Lewis base), which can grab protons from water (H_2_O) molecules to produce hydroxide ions (OH^−^). In other words, concentrated hydroxide ions locally accumulate near the positively charged –NH_3_^+^ groups through electrostatic interaction. As a result, adding RuCl_3_ to the aqueous dispersion of APTES-modified SiO_*x*_ NSs triggers the hydrolysis of Ru^3+^ primarily near the –NH_3_^+^ groups, leading to the formation and condensation of Ru(OH)_3_ on the surfaces of the SiO_*x*_ NSs. The heterogeneous nucleation and growth of Ru(OH)_3_ NPs on the SiO_*x*_ NSs and the localized high concentration of OH^−^ near the SiO_*x*_ NSs accelerate the hydrolysis of Ru^3+^ to complete within 5 s. By contrast, without surface modification of APTES, the hydrolysis of Ru^3+^ cannot rapidly and preferably occur on the surfaces of the SiO_*x*_ NSs due to the low concentration of OH^−^ throughout the reaction solution. Instead, homogeneous hydrolysis slowly proceeds in solution, resulting in the precipitation of black powders at the bottom of the reactor (Fig. S2).

Annealing the Ru(OH)_3_/SiO_*x*_ composite particles at elevated temperatures (e.g., 150 °C) in air dehydrates the Ru(OH)_3_ nanoparticles, forming RuOOH ones by the reaction of Eq. 1:

1 $$ {\text{Ru}}\left( {\text{OH}} \right)_{3}  \mathop{\longrightarrow}\limits^{\Delta }{\text{RuOOH}} + {\text{H}}_{2} {\text{O}} . $$

**Fig. 1:**
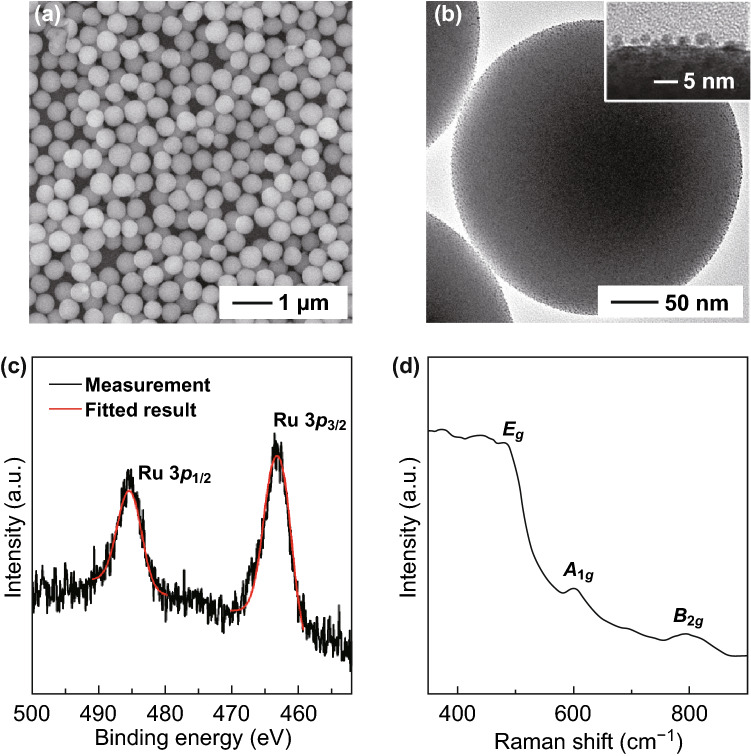
**a** SEM image of RuOOH/SiO_*x*_ composite particles. **b** TEM image of an individual RuOOH/SiO_*x*_ composite particle, showing good dispersion of the RuOOH NPs uniformly distributed on the surface of the SiO_*x*_ NS. The inset of a blow-up TEM image highlights the well-defined and separated RuOOH NPs with diameters in the range of 2–3 nm. **c** Experimentally measured (black) and fitted (red) XPS spectrum of the Ru *3p* peaks for the RuOOH/SiO_*x*_ composite particles. **d** Typical Raman spectrum of the RuOOH/SiO_*x*_ composite particles. (Color figure online)

The dehydration treatment significantly increases the stability and wettability of the nanoparticles toward organic solvents of useful reactions. Figure 1a presents a typical SEM image of the RuOOH/SiO_*x*_ composite particles, showing their intact monodispersity and overall spherical geometry. The TEM images of individual composite particles (Figs. 1b and S1b) reveal that the RuOOH NPs with sizes of 2–3 nm are uniformly anchored to the surface of the SiO_*x*_ NSs. Ru *3p* XPS exhibits *3p*_1/2_ and *3p*_3/2_ features with binding energies of 485.3 and 463.5 eV (Fig. 1c), respectively, confirming that the oxidation state of Ru is 3 [23, 24]. The consistent oxidation state of Ru in the precursor salt (RuCl_3_) and the synthesized RuOOH/SiO_*x*_ composite particles indicates the importance of mild thermal annealing that can stabilize the hydrolyzed nanoparticles but not over-oxidize the metal species. The stoichiometric ratio of O in Ru–O–Ru and Ru–OH is determined from peak fitting of the O 1*s* XPS signal (Fig. S3c), which shows the ratio close to 1:1. Therefore, the reasonable stoichiometric formula of the synthesized Ru-containing NPs is RuOOH [25]. The Raman spectrum of the RuOOH/SiO_*x*_ composite particles exhibits multiple peaks at 482, 602, and 790 cm^−1^, corresponding to the *E*_*g*_, *A*_*1g*_, and *B*_*2g*_ modes of Ru–O stretch vibrations, respectively [26, 27]. The strong Ru–O stretch vibrations indicate the formation of strong covalent bond between Ru and O in the small NPs (inset in Fig. 1b) loaded on the SiO_*x*_ NSs, which is consistent with the transformation of ionic-type Ru^3+^/OH^−^ bond in Ru(OH)_3_ NPs to covalent-type Ru(III)-O bond in RuOOH nanoparticles through dehydrogenation reaction of Eq. 1. The absence of XPS signal near the Cl *2p* binding energy (Fig. S3b) confirms the complete removal of possibly adsorbed Cl^−^ ions from the synthesized composite particles during the centrifuge/washing cycles.

**Fig. 2:**
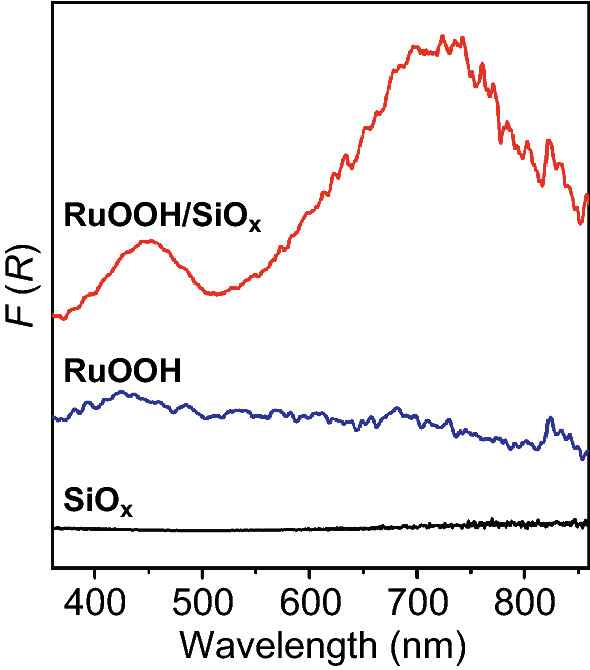
DRS spectra of the synthesized RuOOH/SiO_*x*_ composite particles (red curve), RuOOH powder (blue curve), and SiO_*x*_ NSs (black curve). (Color figure online)

The absorption-sensitive DRS spectrum of the as-synthesized RuOOH powder in the absence of SiO_*x*_ NSs exhibits a broad absorption peak around 430 nm that is attributed to the *p → d* interband transitions (blue curve, Fig. 2) [28]. The two minor sharp peaks at 686 and 825 nm correspond to the Ru *t*_*2g*_ Drude-like intraband transitions. The pristine SiO_*x*_ NSs exhibit essentially no light absorption in the studied spectral region (black curve, Fig. 2). The RuOOH/SiO_*x*_ composite particles shown in Fig. 1a, which are composed of ultrafine RuOOH NPs uniformly dispersed on the surfaces of the SiO_*x*_ NSs, exhibit a DRS spectrum (red curve, Fig. 2) significantly different from the sum of the spectra of both RuOOH powder and the SiO_*x*_ NSs. The light absorption intensity of the RuOOH/SiO_*x*_ composite particles becomes much higher than that of the counterpart pure particles, indicating the role of the SiO_*x*_ NSs in enhancing light absorption in the RuOOH NPs. Such enhancement intensifies the characteristic absorption peaks of the RuOOH nanoparticles although their appearance may be concealed due to the overlap with other newly formed peaks. For example, the Ru *t*_*2g*_ intraband transition peaks are embedded in the new wide/intense peak around 724 nm. This additional strong peak is ascribed to the antenna effect of the SiO_*x*_ NSs on which strong surface light scattering resonances can occur to create significantly enhanced electric fields near the surfaces of the SiO_*x*_ NSs [18]. The ultrasmall RuOOH NPs dispersed on the SiO_*x*_ NSs benefit from the locally intensified electric fields to enhance their light absorption power, showing strong absorption peaks correspondingly.

As discussed in Introduction, the weak light absorption in the highly dispersed small RuOOH NPs limits their use as an efficient photothermal catalyst for the selective aerobic oxidation of BzOH. This challenge is overcome by loading the RuOOH NPs to the SiO_*x*_ NSs because of the antenna effect of the SiO_*x*_ NSs as highlighted in Fig. 2. A white LED lamp with an emission spectrum in the range of 400–700 nm (Fig. S4) has been used to evaluate the photothermal efficiency of the RuOOH/SiO_*x*_ composite particles of Fig. 1a. The typical heating and cooling profile of a BTF dispersion of the RuOOH/SiO_*x*_ composite particles is presented in Fig. S5a (red dots) when the LED lamp of 520 mW (i.e., power intensity of light interacting with the RuOOH/SiOx particles in the test vial) is alternatively turned on and off. At the test condition, the dispersion of the RuOOH/SiO_*x*_ composite particles shows a temperature increase of 23.1 °C at equilibrium under the continuous light illumination, whereas the dispersion of the same amount of pristine RuOOH powder (in the absence of SiO_*x*_ NSs) shows a temperature increase of only 9 °C (Fig. S5a, red dots versus blue dots). The SiO_*x*_ NSs do not show an observable photothermal effect (Fig. S5a, black dots). The comparison confirms that both the small size of nanoparticles of a light-absorbing material and the antenna effect of the SiO_*x*_ NSs are beneficial to high photothermal efficiency.

**Fig. 3:**
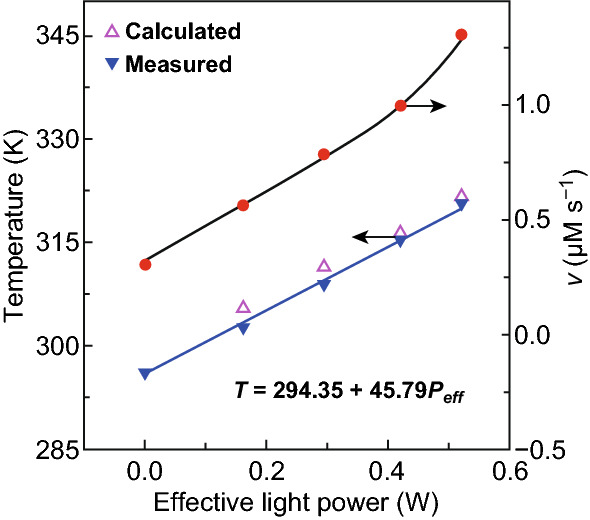
Photothermally induced increase in (triangles) temperature of the reaction solution and (solid red dots) reaction rate in the presence of the RuOOH/SiO_*x*_ composite particles as a photothermal catalyst. Blue triangles indicate the temperature measured by the thermocouple probe under the illumination of different effective light powers (*P*_eff_), showing a linear dependence. Violet triangles correspond to the reaction temperatures calculated from the measured reaction rates (solid red dots) according to the Arrhenius equation. (Color figure online)

Because of the importance of aerobic selective oxidation of BzOH to BzAD and the catalytic activity of the small RuOOH nanoparticles, the selective oxidation of BzOH to BzAD has been chosen to evaluate the efficiency of the RuOOH/SiO_*x*_ composite particles as a new class of photothermal catalyst. In a reaction solution containing a given amount of RuOOH/SiO_*x*_ particles, the equilibrium temperature reached under the illumination of an LED lamp can be tuned by varying the light power density. The solution temperature (*T*) exhibits a linear dependence, *T/*K = 294.35 + 45.79*P/*W, on the effective light power density (*P*_eff_) in the range of 161–520 mW (Fig. 3, blue triangles). The corresponding photothermal energy conversion efficiency of the absorbed light in the RuOOH nanoparticles, *η*_ab*s*_, is close to the unity (or ~ 100%), and the apparent total photo-to-thermal efficiency with respect to the power of light interacting with the RuOOH/SiOx composite particles (*η*_total_) is 92.47% (Figs. S5 and S6).

The reaction rate of BzOH-to-BzAD conversion in the presence of the RuOOH/SiO_*x*_ composite particles is 0.36 μM s^−1^ (Fig. S7, blue dots), corresponding to a turnover frequency (TOF) of 5.2 × 10^−4^ s^−1^ normalized against the number of Ru atoms, at 24 °C in the dark under the atmosphere of 1 atm O_2_. Illuminating the reaction solution accelerates the oxidation reaction along with the increase in temperature (Fig. 3, red dots). For instance, the reaction rate increases to 1.30 μM s^−1^ (or TOF of 1.89 × 10^−3^ s^−1^) under the illumination of effective power of 520 mW (Fig. S7, red dots), highlighting that the photothermally induced temperature increase is indeed capable of accelerating the oxidation of BzOH. To further confirm that the reaction rate is solely improved by the photothermal effect, the oxidation of BzOH reaction is performed at a constant temperature of 35 °C by immersing the reaction vial in a large-volume water bath, while light illumination is alternatively turned on and off. Regardless of the photo-illumination condition, the reaction exhibits a constant reaction rate that is determined from the constant slope of the time-dependent yield of BzAD (Fig. S8). The unchanged reaction rate in the course of the entire oxidation reaction excludes the involvement of hot-electron chemistry that usually significantly boosts reaction kinetics. In contrast, the control experiments in the absence of RuOOH/SiO_*x*_ composite particles or in the presence of only SiO_*x*_ NSs do not show the detectable formation of BzAD (Table S1). Moreover, both the pristine RuOOH powder of large particles (Fig. S9a) and the agglomerated RuOOH NPs of large sizes on SiO_*x*_ NSs (Fig. S9b) cannot catalyze the oxidation of BzOH even though the temperature of the reaction solution elevates under photo-illumination. Comparison with the control experiments indicates that the small size of the RuOOH NPs is crucial to achieving the high catalytic activity, which can be further enhanced by the photothermal effect. The longtime stability has been evaluated using the RuOOH/SiO_*x*_ hybrid particles of Fig. 1 as the photothermal catalyst. No obvious decrease in reaction rate is observed even after the reaction lasts 24 h under photo-illumination (Fig. S10). The TEM images of the RuOOH/SiO_*x*_ composite particles after the 24-h reaction indicate that the composite particles, in particular the small RuOOH NPs, are intact (inset, Fig. S10). The retaining of both the photothermal catalytic activity and the structural geometry/integrity of the RuOOH/SiO_*x*_ composite particles after a longtime reaction highlights the stability of the RuOOH/SiO_*x*_ catalyst.

The activation energy of the selective oxidation of BzOH on the RuOOH NPs is determined as 41.75 kJ mol^−1^ from the dependence of reaction rate in the dark on the solution temperature according to the Arrhenius equation (Fig. S11). Inserting these reaction rates measured under light illumination of varying power densities into the Arrhenius equation, we can estimate the reaction temperatures (Fig. 3, open violet triangles) necessary to support the observed reaction rates. The good agreement between the estimated temperatures and the solution temperatures directly measured with the thermocouple (open triangles versus solid triangles, Fig. 3) indicates that the acceleration of the oxidation reaction in the presence of RuOOH/SiO_*x*_ composite particles under photo-illumination is primarily ascribed to the photothermal effect.

The selective oxidation of BzOH to BzAD in the presence of RuOOH/SiO_*x*_ composite particles strongly depends on the partial pressure of O_2_ (i.e., the reaction rate increases with the partial pressure of O_2_). The observation is significantly different from the previously reported hydrated RuO_2_ and Ru(OH)_3_ catalysts on which the rate-determining step (RDS) of oxidation reactions involves the elimination of β-H of BzOH and is independent of the partial pressure of O_2_ [12–15]. The difference indicates a different reaction mechanism on the ultrasmall RuOOH NPs, where oxygen involves in the RDS of oxidizing BzOH. The freestanding molecular oxygen, O_2_, is inert at mild temperature toward the elimination of β-H of BzOH to drive BzOH-to-BzAD conversion [29, 30]. The coordination number of Ru in the RuOOH solids is 3 and most likely to decrease to 2 on the surface [31–33]. The low coordination number of surface Ru(III) in the small RuOOH NPs leads to the easy adsorption of O_2_ by forming coordination bonds between O_2_ and Ru. Ru(III) possesses the ability to partially donate one electron to the antibonding orbital of the flat-on/end-on adsorbed O_2_, weakening the oxygen–oxygen double bonds to increase the reactivity of adsorbed O_2_. The activated O_2_ reacts with β-H of BzOH to form BzAD. Such reaction mechanism involving the activation of adsorbed O_2_ is supported by fitting the reaction kinetics using the Langmuir–Hinshelwood (L–H) mechanism (Eq. 2) where both BzOH and O_2_ are adsorbed on RuOOH NPs [34, 35]:

2 $$ \nu = \frac{{\gamma K_{1} K_{2} \left[ {\text{BzOH}} \right]\left[ {{\text{O}}_{2} } \right]_{\text{sol}}^{n} }}{{\left[ {1 + K_{1} \left[ {\text{BzOH}} \right] + K_{2} \left[ {{\text{O}}_{2} } \right]_{\text{sol}}^{n} } \right]^{2} }}, $$

where *γ* is the rate coefficient, *K*_1_ and *K*_2_ are the adsorption equilibrium constants defined by the ratio of rates of surface adsorption and desorption for BzOH and O_2_, respectively, which depend on temperature and the surface chemistry of catalysts. The value of *n* can be taken as 1 and 0.5 for associative and dissociative adsorption of O_2_, respectively. The concentration of O_2_ dissolved in the reaction solution is proportional to its partial pressure, $p_{{{\text{O}}_{2} }}$, following the Henry’s law (Eq. 3):

3 $$ \left[ {{\text{O}}_{2} } \right]_{\text{sol}} = \frac{1}{{K_{\text{H}} }}p_{{{\text{O}}_{2} }} . $$

*K*_H_ is a temperature (*T*)-dependent constant determined by Eq. 4:

4 $$ K_{\text{H}} = K_{\text{H}}^{\theta} { \exp }\left[ { - \alpha \left( {\frac{1}{T} - \frac{1}{{T^{\theta } }}} \right)} \right], $$

where $K_{\text{H}}^{\theta}$ and *T*^ϴ^ are the Henry’s constant and temperature at the standard condition and *α* is a constant.

When molecular oxygen is associatively adsorbed on the RuOOH NPs, Eq. 2 can be simplified to Eq. 5:

5 $$ \sqrt {\frac{{p_{{{\text{O}}_{2} }} }}{\nu }} = \frac{b}{\sqrt a } + \frac{c}{\sqrt a }p_{{{\text{O}}_{2} }} , $$

where *a *= *γK*_1_*K*_2_[BzOH], *b* = 1 + *K*_1_[BzOH], and *c *= *K*_2_/*K*_H_. At a given temperature and a high concentration of BzOH, *a*, *b*, and *c* are considered as constants at the early reaction stage when the conversion of BzOH is low (e.g., < 5%). Similarly, for a reaction involving the dissociative adsorption of O_2_, Eq. 2 becomes Eq. 6:

6 $$ \frac{{\sqrt[4]{{p_{{{\text{O}}_{2} }} }}}}{\sqrt \nu } = \frac{b}{\sqrt a } + \frac{c}{\sqrt a }\sqrt {p_{{{\text{O}}_{2} }} } . $$

**Fig. 4:**
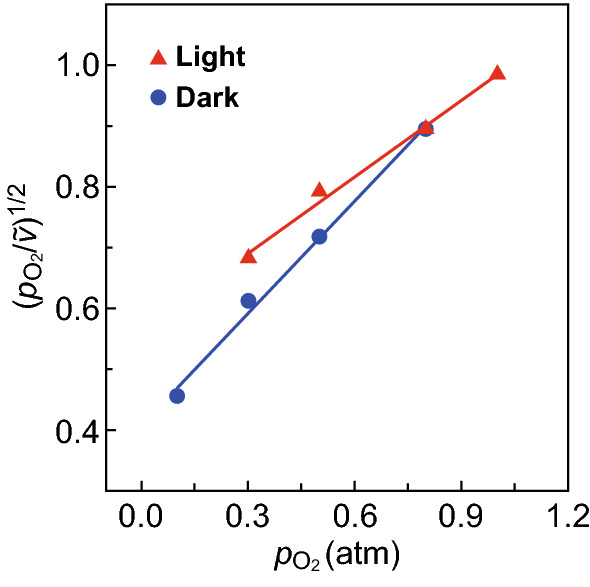
Dependence of reaction rate with a partial pressure of O_2_ ($p_{{{\text{O}}_{2} }}$) in the atmosphere, tested under photo-illumination of an effective light power of 520 mW (red dots) and dark condition (blue dots). The results are plotted according to the linear form of the Langmuir–Hinshelwood model in which oxygen molecules are associatively adsorbed on the catalyst surface. $\tilde{\nu }$ represents the reaction rates normalized against the corresponding reaction rate under 0.8 atm. (Color figure online)

The fitting results shown in Figs. 4 and S13 indicate that oxygen is associatively adsorbed on the ultrasmall RuOOH NPs. The consistent linear dependence of $\sqrt {\frac{{p_{{{\text{O}}_{2} }} }}{\nu }}$ on the partial pressure of O_2_ in the dark and under photo-illumination (Fig. 4 red dots versus blue dots) indicates that photoexcitation of the RuOOH NPs does not change the reaction mechanism. The variation of slope and intercept of the linear fitting for the reaction in the dark and under light is ascribed to the increased temperature under light illumination, which influences the constants, *a*, *b*, and *c*, in Eq. 5. The dependence of the reaction rate on the partial pressure of O_2_ offers a new strategy to control the selective oxidation of BzOH by simply tuning the reaction atmosphere in the presence of the supported ultrasmall RuOOH NPs.

## Conclusion

Ultrafine RuOOH NPs with sizes of 2–3 nm have been successfully synthesized on the surfaces of APTES-modified SiO_*x*_ NSs through controlled hydrolysis of Ru^3+^ ions followed by mild thermal annealing at elevated temperatures. The ultrafine RuOOH NPs are uniformly dispersed on the surfaces of the SiO_*x*_ NSs, exhibiting a high dispersion to expose the large surface area and minimize the usage of Ru as a catalyst. The ultrasmall RuOOH NPs become capable of activating molecular oxygen adsorbed on their surfaces to drive the selective aerobic oxidation of BzOH under ambient conditions, contrast to Ru-containing nanoparticles with larger sizes. The SiO_*x*_ NSs in the RuOOH/SiO_*x*_ composite particles play two essential functions to benefit the composite particles to be an efficient photothermal catalyst. First, the SiO_*x*_ NSs serve as support to maintain the high dispersion of the ultrasmall RuOOH NPs and good colloidal stability even under reaction conditions as shown in this work. Second, the intense surface light scattering resonances on the SiO_*x*_ NSs significantly increase the light absorption power of the RuOOH NPs and thus their photothermal conversion efficiency even with the reduced amount of Ru. Forming the RuOOH/SiO_*x*_ composite particles, for the first time, enables photothermal catalysis of selective aerobic oxidation of BzOH to BzAD with high energy efficiency. The materials’ design and synthesis described in this work open a promising avenue to explore the unique properties of functional NPs enabled by the ultrasmall sizes.

## Electronic supplementary material

Below is the link to the electronic supplementary material.

Supplementary material 1 (PDF 1590 kb)
