# Supplementary material for: Highly Dispersed RuOOH Nanoparticles on Silica Spheres: An Efficient Photothermal Catalyst for Selective Aerobic Oxidation of Benzyl Alcohol
Source: Nanomicro Lett. 2020 Jan 27;12:41. doi: 10.1007/s40820-020-0375-9 (PMC7770779; doi:10.1007/s40820-020-0375-9)
Supplement: Supplementary file 1 — Supplementary material 1 (PDF 1590 kb) [file 40820_2020_375_MOESM1_ESM.pdf]

Supporting Information for

Highly Dispersed RuOOH Nanoparticles on Silica Spheres: An Efficient Photothermal Catalyst for Selective Aerobic Oxidation of Benzyl Alcohol

Qilin Wei[1], Kiersten G. Guzman[1], Xinyan Dai[1], Nuwan H. Attanayake[1], Daniel R. Strongin[1], Yugang Sun[1,] *

[1]Department of Chemistry, Temple University, 1901 North 13th Street, Philadelphia, Pennsylvania 19122, USA

*Corresponding author. E-mail: ygsun@temple.edu (Yugang Sun)

Supplementary Figures and Table

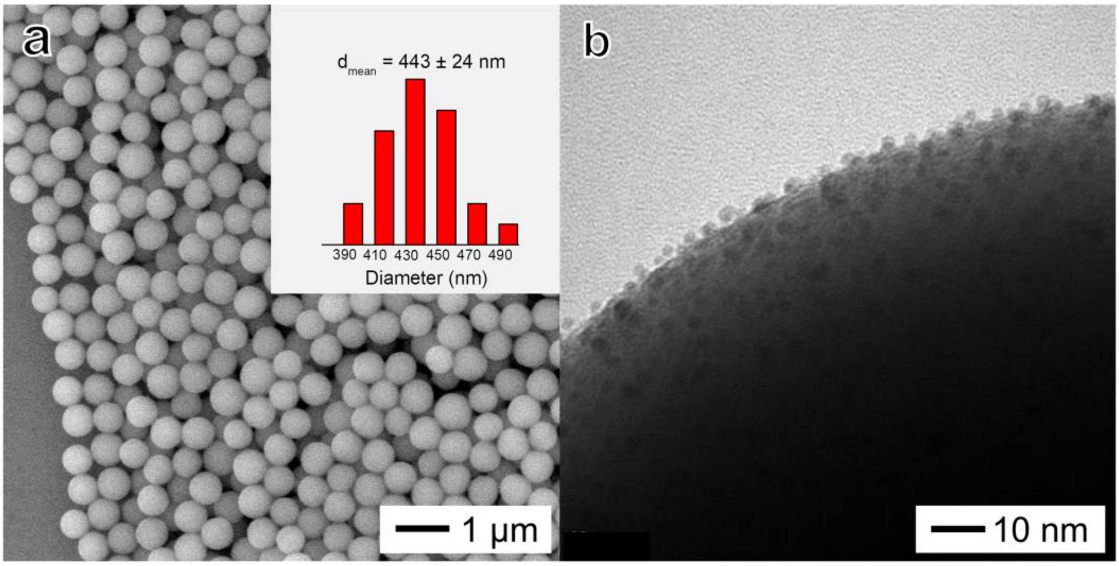

Fig. S1 a SEM image and (inset) dimensional statistic histogram of SiO_x NSs, showing their good monodispersity and narrow size distribution (variation of 5.5 %). **b** TEM image of partial RuOOH/SiO_x composite particle, highlighting the good dispersity of ultrasmall RuOOH NPs on the SiO_x surface

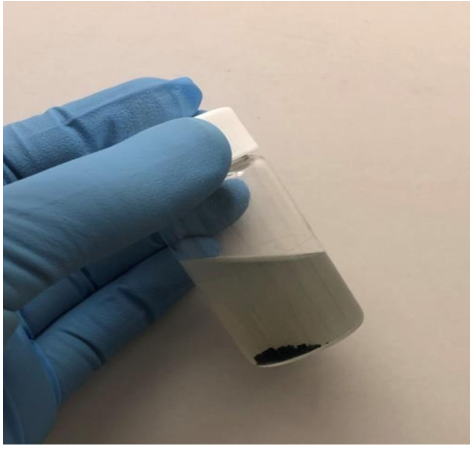

Fig. S2 Digital photograph of the product formed from the slow hydrolysis of $RuCl_3$ in the presence of SiO_x NSs without surface modification. Although the SiO_x NSs are well dispersed, the hydrolysis product of Ru(III) precipitates to the bottom of the reaction vial as black powders

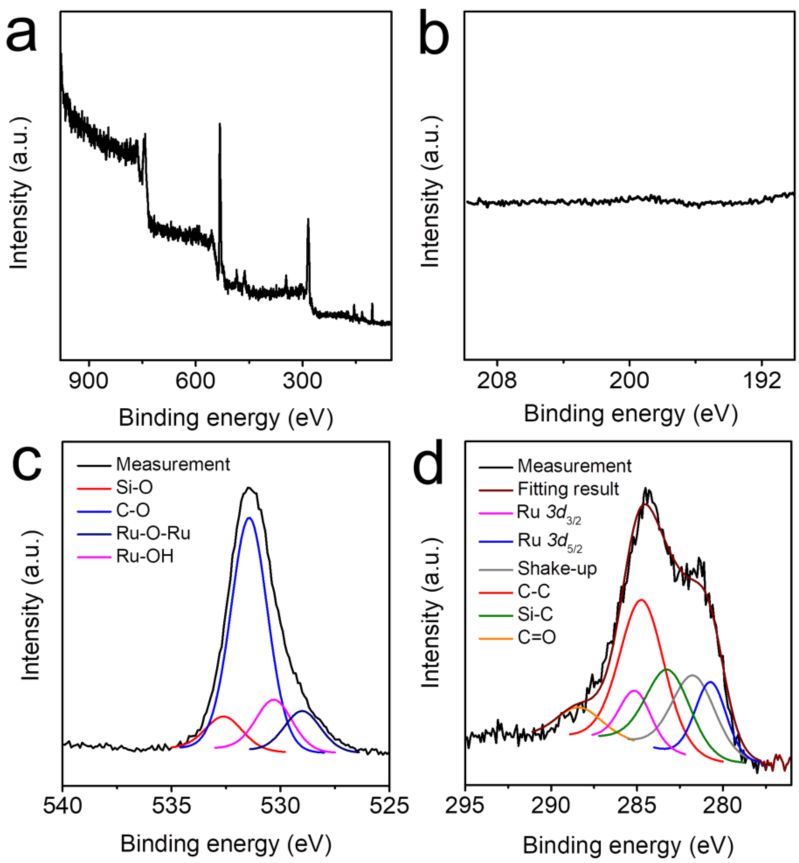

Fig. S3 XPS characterization of the synthesized RuOOH/SiO_x composite particles: **a** survey spectrum, **b** high-resolution spectrum in the energy range of Cl *2p* signal, **c** high-resolution spectrum in the energy range of O *1s* signal, and **d** high-resolution spectrum in the energy range of both C *1s* both Ru *3d* signals

In Fig. S3b, the absence of Cl *2p* signal indicates that the synthesized particles are free of Cl^{-}. In Fig.S3c, two O *1s* peaks centering at 530.3 and 529.0 eV are assigned to oxygen in Ru-OH and Ru-O-Ru, indicating the co-existence of Ru-OH and Ru-O-Ru in the synthesized particles with a ratio of peak areas close to 1:1. Therefore, the reasonable stoichiometric formula of the synthesized Ru-containing NPs is RuOOH.

In Fig. S3d, the Ru *3d* signals of any specific chemical bonds were fitted by following the theoretical spin orbit splitting of 4.2 eV and the area ratio of $3d_{3/2}$ to $3d_{5/2}$ of 2:3. Due to the inevitable organic contaminations, the Ru *3d* signals are interfered by the more intense C *1s* signals. In contrast, the XPS signals of organic contaminations do not overlap with the Ru *3p* peaks, which are presented in Fig. 1c.

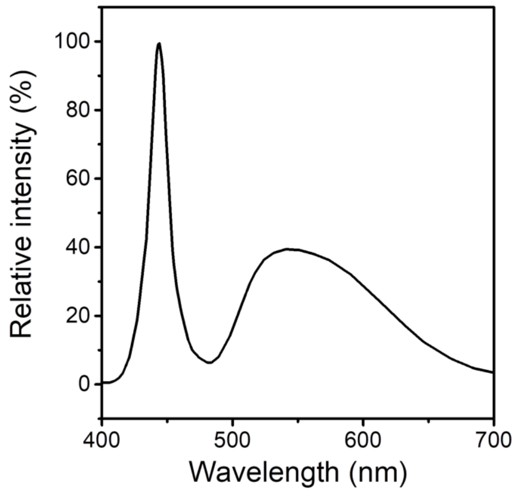

Fig. S4 Emission spectrum of the LED lamp used in the experiment

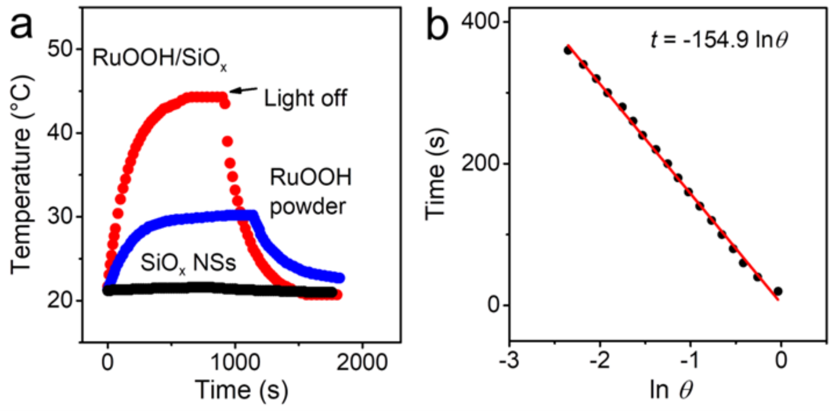

Fig. S5 Photothermal effect of RuOOH/SiO_x composite particles, RuOOH powders and SiO_x NSs. Test conditions: 3 mL of BTF containing 20 mg of RuOOH/SiO_x composite particles (red dots), or same mass of freestanding RuOOH powders (blue dots), or same mass of freestanding SiO_x NSs (black dots); effective light power of 520 mW. The temperature was measured using a thermocouple immersed in the particle dispersions. **a** Time-dependent

variation of temperature when the LED lamp was turned on and turned off. **b** Linear fitting of time (t) and lnθ (θ defined in the following content) according to the equation: $t = -\tau_s \times \ln\theta$ using the data of RuOOH/SiO_x composite particles during the cooling period. The slope determines the value of τ_S.

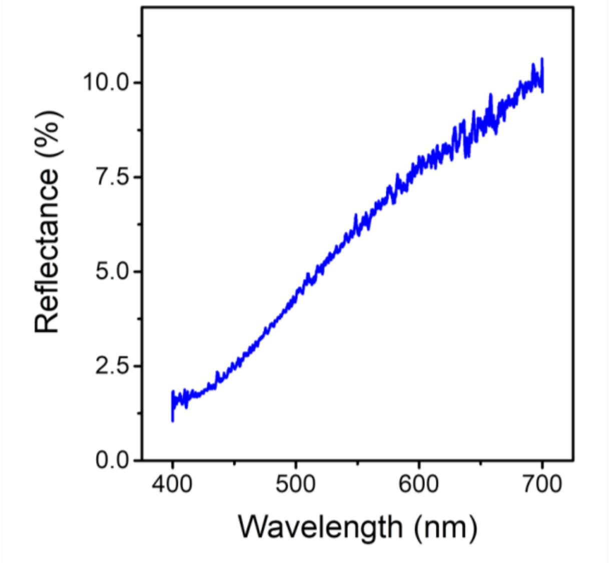

Fig. S6 Reflectance spectrum of the dispersion of RuOOH/SiO_x composite particles in BTF, same as the dispersion used in Fig. S5

The photo-to-thermal conversion efficiency of the absorbed light is calculated according to the protocol described in the previous report [S1]. The efficiency is expressed as Eq. S1:

$$\eta_{abs} = \frac{hS(T_{Max} - T_{Surr}) - Q_{Dis}}{P_{abs}} \qquad \text{(S1)}$$

where h is the heat transfer coefficient from the heated reaction solution and reactor (e.g., glass vial in this work), S is the surface area of the reactor. T_{Max} is the maximum steady temperature enabled by photothermal effect, which was 44.3 °C under the experimental condition of Fig. 3. The surrounding environment temperature, T_{Surr}, was 21.2 °C. Q_{Dis} represents the heat generated by the light absorption and photothermal conversion of the reactor (i.e., glass vial) and solvent, which was trivial in this work since illuminating the blank solvent in the glass vial only rose the temperature by 0.5 °C even with light power density of 965 mW (Fig. S5a, black dots). The light power absorbed by the solution was measured by using a power meter. A dimensionless parameter, θ, is defined and calculated using Eq. S2:

$$\theta = \frac{T - T_{Surr}}{T_{Max} - T_{Surr}} \qquad \text{(S2)}$$

in which T represents the temperature of the solution at a given time (t). The system time

constant τ_s is calculated from Eq. S3:

$$t = -\tau_s \ln(\theta) \qquad \text{(S3)}$$

Fitting experimental data shown in Fig. S5b resulted that the value of τ_s was 154.9 s. The value of hS was determined according to Eq. S4:

$$hS = \frac{mC}{\tau_s} \qquad \text{(S4)}$$

where m is the mass of the solution (3.57 g), C is the heat capacity of BTF with the value of 0.89 $J \cdot g^{-1} \cdot K^{-1}$ [S2]. The contribution of RuOOH/SiO_x composite particles to hS is ignored due to the negligible mass. Therefore, hS is calculated as 20.5 $mW \cdot K^{-1}$, and $hS(T_{Max} - T_{Surr})$ becomes 473.55 mW.

When a particle dispersion is illuminated with a light, the effective incident light powder (P_{eff}) is distributed into various fractions, including diffuse reflection (P_{dr}), both Rayleigh scattering and Mie scattering (P_s), absorption (P_{abs}) and transmission (P_t):

$$P_{eff} = P_{dr} + P_s + P_{abs} + P_t. \qquad \text{(S5)}$$

In the typical experiment, the power of the LED lamp was measured by Thorlabs PM100D equipped with a S305C thermal sensor, showing the value of P_0 = 965 mW. However, the curved surface of the glass vial reactor reflected a significant amount of light, reducing the light reaching to the RuOOH/SiO_x composite particles dispersed in BTF. By measuring the light reflections on the wall of the glass vial reactor, the effective incident light power interacting with the RuOOH/SiO_x composite particles was determined as P_{eff} = 520 mW. The diffusion reflection spectrum of the dispersed RuOOH/SiO_x composite particles is presented in Fig. S6. Because the diffuse reflection coefficient of the RuOOH/SiO_x composite particles is dependent on wavelength and the emission spectrum of the LED lamp also depends on the wavelength, the corresponding power of diffuse reflections is calculated from Eq. S6:

$$P_{dr} = P_{eff} \frac{\int_{400}^{700} R(\lambda) LED(\lambda) d\lambda}{\int_{400}^{700} LED(\lambda) d\lambda} \qquad \text{(S6)}$$

where $LED(\lambda)$ and $R(\lambda)$ are the intensity of LED light (Fig. S4) and the diffuse reflectance at the wavelength, λ, respectively. The calculations resulted P_{dr} = 27.82 mW. The power of the transmitted light was P_t = 16 mW. By blocking the specular reflected and transmitted light with a black tape attach to the reactor, the scattering light coming out from both sides of the reactor was measured, showing P_s = 8 mW. Plugging these values to Eq. S5 led to P_{abs} = 468.18 mW. According to Eq. S1, the photothermal energy conversion efficiency of the absorbed light in the SiO_x NS-supported RuOOH NPs, η_{abs}, is determined as ~100%.

The apparent photothermal energy conversion efficiency normalized against the total light

reaching the RuOOH/SiO_x composite particles is calculated from Eq. S7:

$$\eta_{total} = \frac{hS(T_{Max}-T_{Surr})-Q_{Dis}}{P_{eff}} \times 100\% = 91.1\% \quad \text{(S7)}$$

The calculated η_{total} was cross-checked with other method. It is well known that the relationship between temperature change (ΔT) and thermal power input (Q) into a reactor follows [S3]:

$$Q = hS\Delta T \quad \text{(S8)}$$

Therefore, the maximum equilibrium temperature achieved by photothermal heating can be expressed with a slight modification of Eq. S8:

$$T_{Max} = T_{Surr} + \eta_{total}\frac{P_{eff}}{hS} \quad \text{(S9)}$$

Linear fitting in Fig. 3 in the main text ($T_{Max} \sim P_{eff}$) shows a slope value as 45.79. The η_{total} is then calculated using the measured data (T_{Surr} = 294.35 K, hS = 20.5 mW·K^{-1}), giving the value of 93.9%. The high consistence of η_{total} determined from fitting two different sets of data highlights the fidelity of estimation processes. Averaging the two values of η_{total} results in 92.5%.

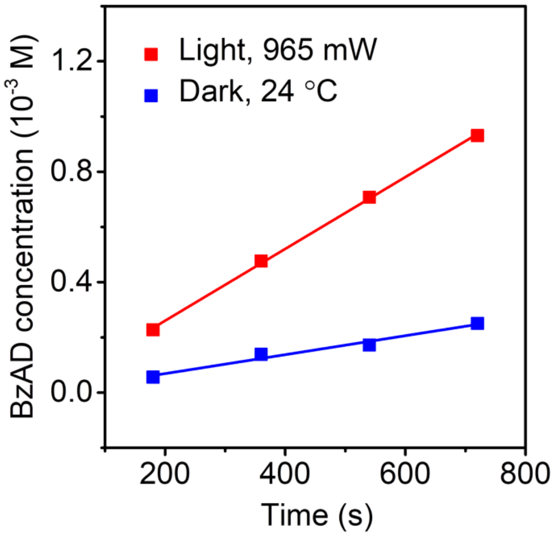

Fig. S7 Time-dependent yield of BzAD generated from the catalytic oxidation of BzOH in the presence of the RuOOH/SiO_x composite particles as catalyst under different reaction conditions: (blue) dark and room temperature (24 °C) and (red) photo-illumination of visible light with power of 965 mW. The slopes of the fitted linear lines represent the reaction rates, highlighting that photo-illumination indeed accelerates the selective oxidation of BzOH to BzAD

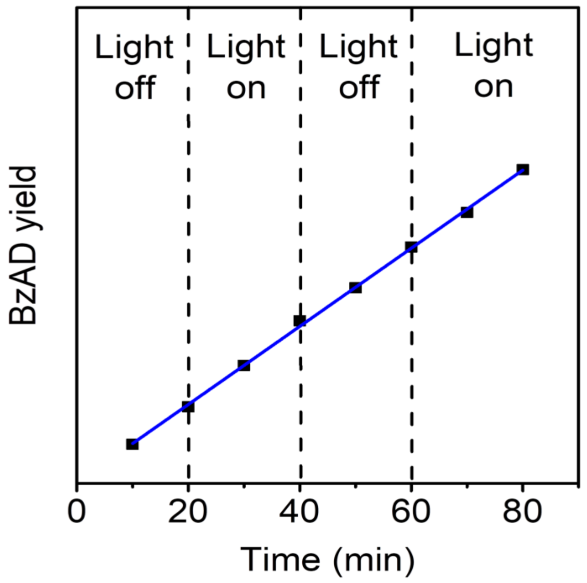

Fig. S8 Time-dependent yield of BzAD generated from the oxidation of BzOH by maintaining the temperature of reaction solution at a constant value, i.e., 35 °C, while light illumination was alternatively turned on and off. The constant temperature was achieved by immersing the reaction vial in a large-volume water bath set at 35 °C. The light power was 965 mW. Regardless of the photo-illumination condition, the constant slope in the course of the oxidation reaction indicates that hot-electron chemistry is absent in the aerobic oxidation of BzOH to BzAD.

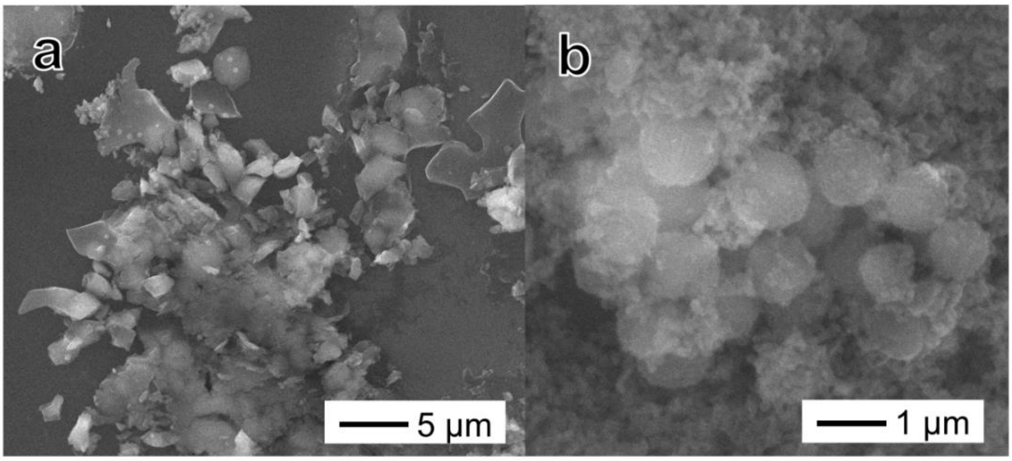

Fig. S9 SEM images of **a** freestanding RuOOH powders synthesized in the absence of SiO_x NSs and **b** RuOOH/SiO_x composite particles synthesized through the simultaneous hydrolysis of Ru(III) and high-concentration of APTES

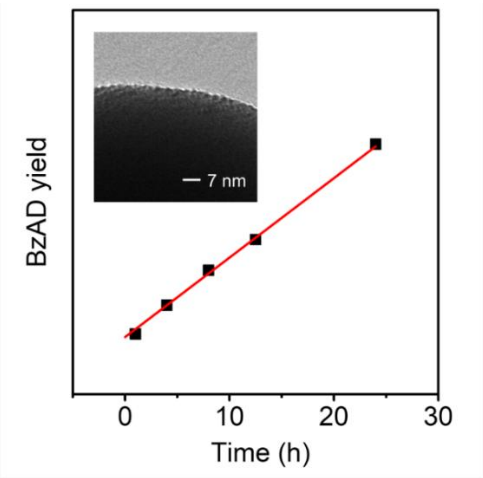

Fig. S10 Long-term stability of the RuOOH/SiO_x composite particles under reaction condition. Reaction condition: 3 mL of BTF containing 3 mg of RuOOH/SiO_x composite particles; 138 μL of BzOH; effective light power of 300 mW. A high concentration of BzOH was chosen to maintain the pseudo zeroth reaction order. The high-quality linear fitting indicates that the reaction rate was maintained in the period of 24 h. The inset shows the TEM image of a RuOOH/SiO_x composite particle after 24-h reaction, highlighting the intactness of the ultrafine RuOOH NPs.

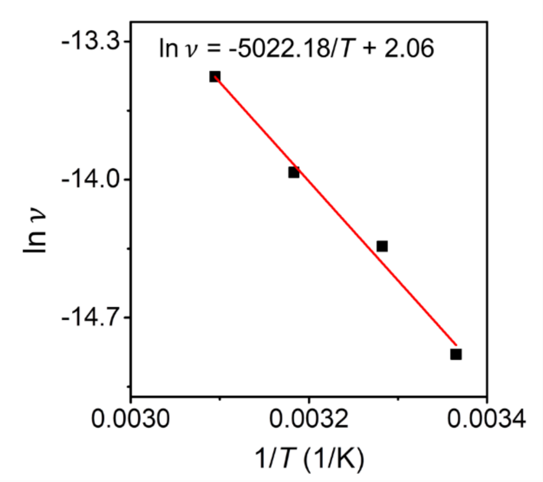

Fig. S11 Dependence of reaction rate (ν, determined by the production rate of BzAD) and the reaction temperature (T) in the dark condition. The reaction rate was determine at the very early stage of the reaction (i.e., the conversion of BzOH was below 1%). The concentrations of reactants, i.e., BzOH and O_2, were considered as constants. Therefore, the reaction rate is proportional to the reaction rate constant (k). The apparent activation energy (E_a) of the reaction were determined by fitting the experimental data according to the Arrhenius equation: $\ln\nu \propto \ln k = \ln A - \frac{E_a}{RT}$ in which A is pre-exponential factor, and R is the universal gas constant with a value of 8.314 J $mol^{-1}\cdot K^{-1}$. The fitting line (red) gives the value of E_a as 41.75 kJ mol^{-1}.

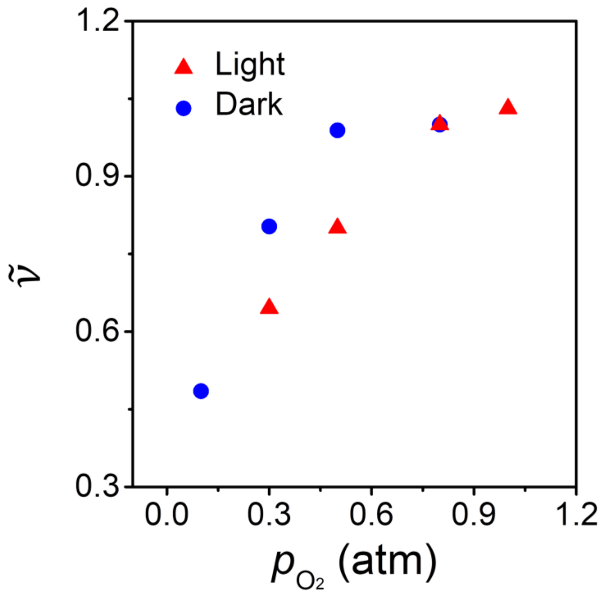

Fig. S12 Dependence of reaction rate versus partial pressure of O_2 (p_{O_2}) under light condition (red dots) and dark condition (blue dots). $\tilde{v}$ represents the reaction rate normalized to the corresponding reaction rate at p_{O_2} of 0.8 atm

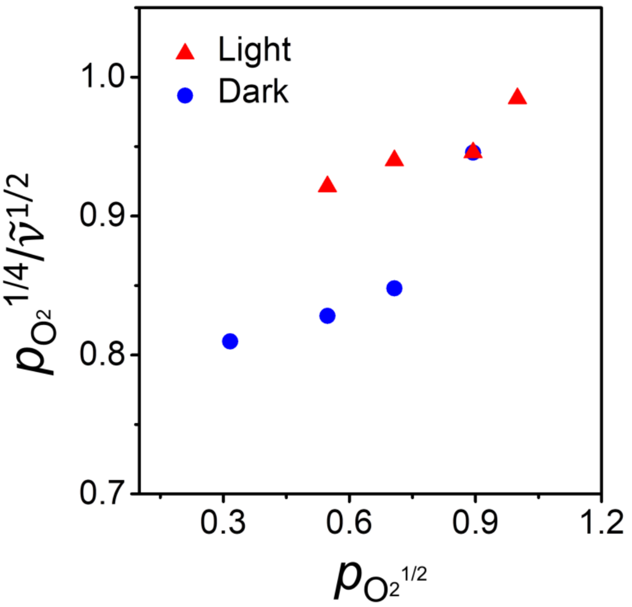

Fig. S13 Dependence of reaction rate versus partial pressure of O_2 (p_{O_2}) under light condition (red dots) and dark condition (blue dots). $\tilde{v}$ represents the reaction rate normalized to the corresponding reaction rate at p_{O_2} of 0.8 atm. The data is plotted according to the linear form of Langmuir-Hinshelwood model where oxygen molecules are dissociatively adsorbed. The non-linear dependence shown in the plots indicates that adsorbed oxygen on the surfaces of the RuOOH NPs does not follow the dissociative activation mechanism.

Table S1 Results of control experiments

Experiment conditions	BzAD yield (mmol)
In the absence of RuOOH/SiO_x composite particles, with light illumination	0
In the presence of APTES-modified SiO_x NSs, without RuOOH NPs, with light illumination	0
In the presence of RuOOH powder only, with light illumination	0
In the presence of RuOOH powder/SiO_x composite particles of Fig. S9b, with light illumination	0

Supplementary References

[S1] X. Liu, B. Li, F. Fu, K. Xu, R. Zou et al., Facile synthesis of biocompatible cysteine-coated CuS nanoparticles with high photothermal conversion efficiency for cancer therapy. Dalton Trans. **43**, 11709 (2014). https://doi.org/10.1039/c4dt00424h

[S2] D.W. Scott, D.R. Douslin, J.F. Messerly, S.S. Todd, I. A. Hossenlopp, T.C. Kincheloe, J. P. McCullough, Benzotrifluoride: chemical thermodynamic properties and internal rotation. J. Am. Chem. Soc. **81**, 1015 (1959). https://doi.org/10.1021/ja01514a001

[S3] D.K. Roper, W. Ahn, M. Hoepfner, Microscale heat transfer transduced by surface plasmon resonant gold nanoparticles. J. Phys. Chem. **111**, 3636 (2007). https://doi.org/10.1021/jp064341w
